# Is there a relationship between executive functions and resilience in youth elite soccer players?

**DOI:** 10.1002/brb3.3122

**Published:** 2023-08-25

**Authors:** Jennifer Lehmann

**Affiliations:** ^1^ Institute of Sport Science, Faculty of Human Sciences University of Regensburg Regensburg Germany

**Keywords:** executive functions, resilience, youth elite soccer players

## Abstract

**Background:**

While the relationship between cognitive performance and sport performance in youth elite soccer players has proponents as well as opponents, many aspects of this relationship remain unclear. Therefore, this quasi‐experimental study wants to contribute to this relationship including the psychological aspect of resilience when investigating youth elite soccer players during an assessment selection for a representative team.

**Methods:**

Questionnaires as well as computer‐based tests were conducted.

**Results:**

Results of this study showed no relationship between resilience and executive function in youth elite soccer players. Furthermore, no differences in executive functions or in resilience were found between those players who are selected for a representative team and those who were not selected.

**Conclusion:**

The results indicate that further research needs to be conducted to clarify possible relationships in more detail.

## Graphical abstract

The article addresses the relationship between cognitive performance and resilience in youth elite soccer players. Thereby, it is investigated whether there are differences between those players who are selected for a squad compared to those players who are not selected.

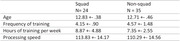

## 1 INTRODUCTION

The transition of soccer during the last decades has increased the cognitive prerequisite in players. This is reflected in research that progressively addresses these aspects not only in adult soccer players (e.g., Vestberg et al., 2012) but also increasingly in youth soccer players (Huijgen et al., 2015; Verburgh et al., 2014; Vestberg et al., 2017). Recent meta‐analyses support the relation between cognitive abilities and sport performance (Mann et al., 2007; Scharfen & Memmert, 2019; Voss et al., 2010), showing small to medium effect sizes regarding superior cognitive skills in elite athletes compared to nonelite athletes (Formenti et al., 2022). On the contrary, others claim that there is no such relationship between cognitive performance and sports expertise (Beavan et al., 2020; Kida et al., 2005; Lundgren et al., 2016).

Cognition that is relevant for team sport is often connected to quick assessment of situations and the appropriate adaption of strategies to these situations, including inhibition of specific responses. These cognitive abilities are subsumed under the concept of executive functions (EF), which develop before early adolescence (Luciana et al., 2005).

The cognitive construct of EF consists of the core skills described as working memory, inhibition, and cognitive flexibility (Diamond, 2013; Miyake et al., 2000). Working memory refers to the ability to hold and mentally manipulate information, while inhibition is the ability to control one's attention, behavior, and thoughts in such a way as to override an intrinsic action in favor of a more appropriate one (Diamond, 2013). Furthermore, cognitive flexibility comprises the ability of perspective changing as well as the adjustment to changing demands (Diamond, 2013). These interrelated skills lead to higher order cognitive functions such as reasoning and problem‐solving (Collins & Koechlin, 2012; Lunt et al., 2012). In the context of sport, EF are required for inter alia the interaction with their match environment (Jacobsen & Matthaeus, 2014).

EF have been investigated in adult elite soccer players (e.g., Vestberg et al., 2012) showing that high‐division soccer players outperform low‐division soccer players, and both groups outperform a norm group. This indicates that cognitive functions are predictive of the success of soccer players. Moreover, Vestberg et al. (2017) were able to extend these previous findings from adult soccer players to young elite soccer players. They found that compared to nonelite players, youth elite soccer players have higher EF, which are relevant for success in soccer players. This is in line with the results of Verburgh et al. (2014), who compared highly talented youth soccer players with matched amateur soccer players in a stop signal task, the Attention Network Test, and a visuospatial working memory task. The highly talented youth soccer players showed better results in the stop signal task, described as motor inhibition, compared to the amateur soccer players. Additionally, Huijgen et al. (2015) compared elite youth soccer players with subelite youth soccer players regarding their cognitive functions. Elite youth soccer players showed better results in inhibitory control, cognitive flexibility, and metacognition when compared to subelite youth soccer players. Sakamoto et al. (2018) demonstrated the importance of executive functions, alongside physical performance, for admission to an elite youth program in Japanese soccer. However, this context has yet to be studied in a European context. Thus, this study aims to investigate whether executive functions should be considered in talent scouting for youth soccer players in Europe.

Although most of the aforementioned studies have been cross‐sectional, there is clearly a need for longitudinal studies. One longitudinal study regarding the executive functions in high‐level soccer players was recently conducted by Beavan et al. (2020). They investigated the development of EF in a high‐level soccer sample and its relationship to training that those players were exposed to throughout their assessment. While they confirmed the predominant development of EF between the age of 10 and 15 years, they could not show differences between the development trajectories of soccer players and the general population. Therefore, Beavan et al. (2020) raised questions about the relevance of EF in soccer and specifically, in talent scouting in youth elite soccer players. Due to different results in the outcome of the aforementioned studies, Scharfen and Memmert (2021) tried to address these aspects in a more multidisciplinary context, including physiological aspects, game time, game intelligence, and injury aspects with EF in soccer players. Their results revealed, among other things, a relationship between EF and soccer performance, showing that better performance in EF is associated with measured game time that indicates successful soccer performance. Moderate to large correlations were shown for coach‐rated game intelligence and cognitive flexibility to game time. Small to moderate correlations were shown for working memory capacity, cognition score, and selective attention to game time. Additionally, specific physical performance aspects, namely, sprint and a specific endurance aspect (repeated intense exercise ability), contribute to soccer performance (measured with game time). While this research addresses a more holistic approach to identifying relevant aspects, such as cognitive–athletic performance concerning game time, game intelligence, executive functions (working memory capacity, cognitive flexibility, inhibition), and physiological measurements in youth soccer athletes and their success, many questions remain open. Therefore, Scharfen and Memmert (2021) recommend the inclusion of psychological aspects, such as resilience, in this line of research to receive an even more holistic approach. This is what the presented study aims to address in the research question.

The inclusion of psychological aspects in the context of youth soccer players is even more relevant when considering a wide range of pressure that young athletes face in order to achieve a high‐performance level and potentially become professional soccer players. One crucial aspect in this context is resilience, which refers to an individual's ability to cope with negative experiences in such a way that they demonstrate more positive outcomes (Parsons et al., 2016). According to Wagnild and Young's (1993) definition, resilience moderates the negative effects of stress as a personality characteristic that leads to adaptions. Although resilience is often seen as a trait concept, Parsons et al. (2016) argue that there may be other approaches to resilience as well. Based on various aspects concerning the involvement of executive functions in the processes of resilience (Schäfer et al., 2015; Schwager & Rothermund, 2013; Thoern et al., 2016), they propose a cognitive model for resilience that includes executive functions. This model contributes to resilience through both executive control processes and information processing. The differentiation between resilience as a trait or a process is further supported by other research (see Fletcher & Sarkar [2013] for review). While most research on resilience has focused on individuals who have had to react to stressful live events (compare Fletcher & Sarkar, 2012), there is a growing body of research that addresses the concept of stress and resilience in the context of sports.

To date, only a few studies have investigated resilience in the context of soccer specifically. Holt and Dunn (2004) have stated that resilience plays an important role in the process of becoming a high‐level‐performance soccer player. Secades et al. (2017) have shown that athletes with higher resilience scores experience lower levels of stress. However, this has not yet been studied in youth soccer players, particularly in the context of player selection for representative teams as a first step toward becoming a professional soccer player.

While the studies mentioned above have shown different approaches to executive functions, performance, and psychological aspects for elite and nonelite soccer players, none of them have addressed the correlation between executive functions and resilience in single study. Additionally, although these studies differentiate between elite and nonelite soccer players, none have investigated executive function and resilience in a squad selection sample. Therefore, this study aims to contribute to the understanding of executive functions and the role of resilience in a squad selection sample. Specifically, it investigates whether there are detectable differences between youth elite soccer players who are selected for a representative team and those who are not.

Therefore, the following hypothesis were formulated:

1. Executive functions in youth elite soccer players are correlated with resilience.
2. Youth elite soccer players selected for a representative team exhibit better coping mechanisms for stress than nonselected players, resulting in higher resilience scores.
3. Youth elite soccer players selected for a representative team exhibit better executive functions compared to nonselected players (Huijgen et al., 2015).

## 2 METHOD

### 2.1 Participants

Fifty‐nine youth elite soccer players of a German junior performance center participated in this study. The participants were male players aged between 12 and 13 years (*M* = 12.76, *SD* = 0.43). All players played soccer as their main sport at a competitive level. All of them played at state (*n* = 53) or district (*n* = 6) level. Participants were tested during an assessment, where they were evaluated regarding their soccer performance and chosen for a representative team. While the representative team comprised 24 youth elite soccer players, the nonrepresentative team comprised 35 youth elite soccer players. There were no significant differences between the two groups in terms of age (*F*(1,57) = 1.01, n.s.), frequency of training (*F*(1,57) = 1.58, n.s.), hours of training per week (*F*(1,57) = 2.42, n.s.), or processing speed (*F*(1,57) = 0.864, n.s.). The absolute values are presented in Table 1.

**TABLE 1:** Demographical data for the two different groups.

	Squad *N* = 24	Nonsquad *N* = 35
Age	12.83 ± 0.38	12.71 ± 0.46
Frequency of training	4.15 ± 0.90	4.57 ± 1.48
Hours of training per week	8.87 ± 4.88	7.35 ± 2.55
Processing speed	113.83 ± 14.17	110.29 ± 14.56

### 2.2 Measures

#### 2.2.1 Demographical questionnaire

In the self‐generated demographical questionnaire, personal information was collected along with details about participant's athletic career in soccer. We also asked for information about the participant's soccer league, the intensity of their involvement in the sport in terms of hours per week, and the amount of time spent in training for soccer each week.

#### 2.2.2 Processing speed

Cognitive processing speed was assessed using the Number Connection Test (Oswald, 2016), which is equivalent to the Trail Marking Test (Reitan, 1956). In this test, participants are required to connect presented numbers in ascending order as quickly as possible. The test consists of four sheets of paper and two practice examples. The practice sheets comprise the numbers 1–20 in random order, whereas the test sheets consist of the numbers 1–90 in random order. The test was conducted as a group test, with 30 s allowed for each sheet. The achieved numbers were then converted into IQ‐distribution scores using test norms. The correlation between processing speed, the number connection test, and standard IQ test varies between *r* = .60 and *r* = .80 (Vernon, 1993) and the internal consistency as well as the 6‐month test–retest reliability is about *r* = .90–.95.

#### 2.2.3 Resilience

To assess psychosocial stress resistance, the German short version of the Resilience Scale RS‐11 (Schumacher et al., 2005), which is based on the scale developed by Wagnild and Young (1993), was utilized. Therefore, participants were asked to rate 11 attributes using a 7‐point rating scale ranging from 1 (*I do not agree*) to 7 (*I completely agree*).

#### 2.2.4 Executive functions

The core executive functions, updating, inhibition, and cognitive flexibility, were analyzed with the following tasks: the 2‐back task, the flanker task, and the switching task. All three tasks were administered using Open Sesame, an experimental software program, on a 15‐inch laptop. The screen was positioned approximately 50 cm in front of each participant.

##### Two‐back task

Based on the *n*‐back task, which measures working memory and working memory capacity (Kirchner, 1958), the 2‐back task is a specific form of this measurement also known as updating. Each participant was presented with a sequence of letters presented and had to indicate whether the current letter matched the letter presented two letters before by performing a right mouse click. If the letters did not match those presented before, no reaction was required. Each letter was presented for 500 ms, and the next letter appeared after 2500 ms, regardless whether a reaction was needed or not. The tests consisted of one practice block comprising 10 trials with feedback and three blocks of 50 experimental trials without feedback, consisting of 10 targets and 40 distractors each. Reaction time (RT) and accuracy were recorded for the target items.

##### Flanker task

With the flanker task (Eriksen & Eriksen, 1974), inhibition was measured. This is the ability to suppress a possible reaction with regard to presented stimuli. During the task, participants were shown letter combinations, where the middle letter was flanked by three other letters on each side. Participants were instructed to respond by pressing the left mouse key when the letter in the middle was an H or K, or by pressing the right mouse key if the letter in the middle was an S or C. The possible flanking letters were H or K and S or C, resulting in congruent trials if the middle letter was the same as the flanking letter, and incongruent trials when the middle letter was different from the flanking letter (e.g., flanking letter H or K resulted in a congruent condition when the middle letter was also H or K, and in an incongruent condition when the middle letter was S or C). In addition, A and P were used as flanking letters, resulting in a neutral condition. This resulted in 24 different tasks (eight in each condition) and a total of 96 trials. Each trial remained on the screen until a response was given, and 500 ms after the response the next trial appeared. RT and accuracy were recorded. Before the experimental trials, participants completed 10 practice trials that provided feedback. No feedback was given throughout the test.

##### Switching task

Cognitive flexibility, specifically shifting, was assessed using the adapted number–letter task from Rogers and Monsell (1995), as described in Miyake et al. (2000). In this task, four quadrants were presented on a computer screen. When a number–letter pair (e.g., 5E) was presented in one of the four quadrants, participants had to respond in different ways depending on the location of the pair. If the pair was presented in the top two quadrants, participants were instructed to indicate whether the number was odd or even (2, 4, 6, and 8 for even; 3, 5, 7, and 9 for odd). If the pair was presented in the bottom two quadrants, participants were instructed to indicate whether the letter was a consonant or a vowel (G, K, M, and R for consonant; A, E, I, and U for vowel). The task consisted of three blocks. In the first block, the number–letter pairs were presented only in the top two quadrants for 32 target trials. In the second block, the number–letter pairs were presented only in the bottom two quadrants for 32 target trials. In the third block, which consisted of 128 target trials, the number–letter pairs were presented in a clockwise rotation around all four quadrants. Half of the trials in the third block required shifting operations, while the other half did not require any task switching. Participants received 12 practice trials at the beginning of each block with feedback, whereas the main trails did not include feedback. Throughout all trials, they had to respond by button press. In‐between stimuli time after response was 150 ms. The shifting cost was calculated as the difference between average RTs of the first two blocks and the average RTs in the third block, where a shift was necessary.

### 2.3 Experimental procedure

The study employed on a quasi‐experimental design.

### 2.4 Data analyses

RT data were trimmed for outliers throughout all executive functions’ tests. RTs more than 2 *SD*s below or above the mean per condition and per subject were excluded. Respectively, a multiple analysis of variance, Bonferroni corrected, was performed for the dependent variables of working memory and inhibition, shifting as well as for resilience for both divisions. Furthermore, a multiple bivariate correlational analysis was conducted to examine the relationship between resilience and the three components of the executive functions. To account for multiple testing, the significance level was Bonferroni corrected. The data were analyzed using IBM SPSS Statistics V28.0 (Statistical Package for the Social Sciences). A normality check was performed, and the data were found to be normally distributed.

## 3 RESULTS

### 3.1 Resilience

When analyzing the total score of the resilience scale, no significant differences were found between the representative team members and the nonrepresentative team members (*F*(1,55) = 0.122, n.s., *η* ^2^ = .002).

### 3.2 Inhibition

The three conditions in the inhibition task (congruent, incongruent, and neutral) did not differ between the two groups regarding the RT, nor the accuracy rate, as presented in Table 2.

**TABLE 2:** Results of the Flanker test, differentiated between congruent, incongruent, and neutral trials for each comparison.

	Squad–nonsquad
Congruent reaction time	*F*(1,55) = 1.938 , n.s., *η* ^2^ = .034
Accuracy rate	*F*(1,55) = 0.190, n.s., *η* ^2^ = .003
Incongruent reaction time	*F*(1,55) = 0.700, n.s., *η* ^2^ = .013
Accuracy rate	*F*(1,55) = 0.351, n.s., *η* ^2^ = .006
Neutral reaction time	*F*(1,55) = 0.501, n.s., *η* ^2^ = .009
Accuracy rate	*F*(1,55) = 0.013, n.s., *η* ^2^ = .000

*Note*: n.s. = not significant.

### 3.3 Working memory

Results of the analysis of the target RT in the working memory task did not reveal any significant differences between the groups (*F*(1,55) = 1.254, n.s., *η* ^2^ = .022). The same results were found for the target accuracy rate (*F*(1,55) = 0.058, n.s., *η* ^2^ = .001).

### 3.4 Shifting

Furthermore, no significant differences were found for the shifting costs between the two groups (*F*(1,55) = 0.007, n.s., *η* ^2^ = .000).

### 3.5 Correlational analyses

No relationship between the total score of the resilience scale and any tests of the three executive functions was found. This accounts for both comparison of the representative team as well as for the analysis over all participants. The results are presented in Table 3.

**TABLE 3:** Correlational analyses of resilience and executive functions overall and separated for the squad and nonsquad participants.

	1 Flanker Con_RT	2 Flanker Con_AR	3 Flanker Incon_RT	4 Flanker Incon_AR	5 Flanker Neu_RT	6 Flanker Neu_AR	7 Shifting cost	8 WM_RT	9 WM_AR
Resilience									
Overall	.179	–.060	.114	–.056	.167	–.082	.098	.100	.001
Squad	–.079	–.066	.009	–.166	.120	–.097	.349	–.146	.013
Nonsquad	.243	–.061	.154	.001	.185	–.077	–.056	.224	.000

*Note*: For the Flanker test, the three different conditions (congruent, incongruent, and neutral) are presented.

Abbreviations: AR, accuracy rate; RT, reaction time; WM, working memory.

## 4 DISCUSSION

The results of the study indicate that there are no correlations between executive functions and resilience in youth elite soccer players. Furthermore, no significant differences were found for either executive functions or resilience when comparing players who were selected for a representative team with those who were not selected.

While previous research conducted by Huijgen et al. (2015), Verburgh et al. (2014), and Vestberg et al. (2017) demonstrated differences in executive functions between highly talented soccer players and amateur soccer players, this study did not find such differences between youth elite soccer players regardless of whether they were selected for a representative team or not. Verburgh et al. (2014) investigated a similar age range as this study but differentiated between highly talented soccer players and amateur soccer players, whereas all participants in this study were already playing in a regional representative team. Therefore, the lack of differences found in this study may be due to the already high level of all participants. It is possible that cognitive differences may develop at a later stage of developmental. However, Huijgen et al. (2015) did find differences in inhibitory control, cognitive flexibility, and metacognition between elite and subelite soccer players. Although their sample was slightly older (13–17 years of age) than the participants in this study (12–13 years of age), the differences between the studies may be due to the developmental level of the samples and the different levels of soccer players investigated. These findings are consistent with Vestberg et al. (2017), who showed that cognition can predict success in young soccer players aged 12–19 years. As the participants in this study were slightly younger than those evaluated by Vestberg et al. (2017), these effects may be more detectable at a later developmental stage. This is in accordance with the developmental trajectories described by Huizinga et al. (2006), which show that working memory, shifting, and inhibition develop until 15 years of age. They were able to demonstrate that adult level was reached between 11 and 15 years of age in these executive functions. These developmental trajectories are further confirmed by Beavan et al. (2020). Additionally, they found no differences in executive functions between soccer players and the general population trajectories. Therefore, they raise the question of whether executive functions should be included in talent identification for soccer players. So far, the results of this study support Beavan et al. (2020) in advocating for the inclusion of executive functions in the selection process in youth elite soccer players.

Results revealed no differences in resilience or executive functions between the selected or not selected players. Additionally, no relationship between resilience, and therefore the procession of stress, and increased or decreased cognitive functions, in terms of the executive functions, was found. This was true for both the selected and nonselected players, as well as the total sample. As a result, none of the prepared hypotheses could be confirmed.

Based on these findings, the selection of the representative team in this sample may be more influenced by physiological parameters than cognitive aspects during soccer gameplay. This finding is partially consistent and partially contradictory to the results of Scharfen and Memmert (2021), who discovered a relationship between EF and soccer performance in their sample of participants aged 12–34 years. They discovered that better performance in EF was linked to successful soccer performance, as measured by game time. However, they also discovered that physiological parameters, such as sprint and RIEA, were important factors for success in the younger age group. This indicates that a multidisciplinary approach is necessary to comprehend the interplay of various factors in the development of successful soccer players. Taken together, the results of Beavan et al. (2020), Scharfen and Memmert (2021), and this study suggest that physiological parameters are more significant in talent identification than cognitive aspects. Moreover, due to the lack of a comprehensive understanding of the interplay between cognition and these physiological aspects, additional multidisciplinary research is required to clarify this issue.

The aim of this study was to contribute to the complex understanding of cognition and resilience in youth elite soccer players. While only few studies have investigated the influence of resilience on youth soccer players, those studies indicate that resilience is important in the process of becoming a successful soccer player (Holt & Dunn, 2004) and reduces stress factors (Secades et al., 2017). Therefore, this study addressed this aspect in youth elite soccer players but did not find any differences between the players selected for a representative team compared to the nonselected players. This might be due to the selected sample. While previous research has addressed elite to sub‐ or nonelite players, this study investigated elite youth players at a stage where the next selective process for a representative team took place. Compared to the default for the resilience scale used by Schumacher et al. (2005), the participants in this study showed slightly higher mean scores, suggesting that this sample is selective and only more resilient players have been included in the performance center and in this investigated sample. This finding needs to be further investigated in future research. Alongside the nonsignificant results presented in this study, it is important to incorporate physical, cognitive, and combined requirements in the training of these athletes. This could involve including physical tasks with cognitive aspects or tasks that enhance the resilience of the youth soccer players.

### 4.1 Limitation

The age range of the sample selected for this study may have had an impact on the results. Due to the developmental trajectories, future studies should consider including additional age groups. Moreover, the restrictions imposed in the past 2 years due to the COVID‐19 pandemic may have influenced the results. Soccer players were limited in their training during these times, and the development may differ from previous studies that conducted testing before the pandemic. While this is speculative, it could also have an impact on the results.

## 5 CONCLUSION

Although this study is one of the first to examine the psychological aspect of resilience in youth elite soccer players in conjunction with executive functions, no relationship was found between these two concepts. Further research is needed in this area to obtain a more comprehensive understanding when working with youth elite soccer players in the complex setting of becoming elite soccer players.

## CONFLICT OF INTEREST STATEMENT

The author declares no conflicts of interest.

### PEER REVIEW

The peer review history for this article is available at https://publons.com/publon/10.1002/brb3.3122

## Data Availability

The data that support the findings of this study are available on request from the corresponding author. The data are not publicly available due to privacy or ethical restrictions.
